# Supplementary figures and images for: Dengue seroprevalence study in Bali
Source: PLoS One. 2023 Jul 14;18(7):e0271939. doi: 10.1371/journal.pone.0271939 (PMC10348525; doi:10.1371/journal.pone.0271939)

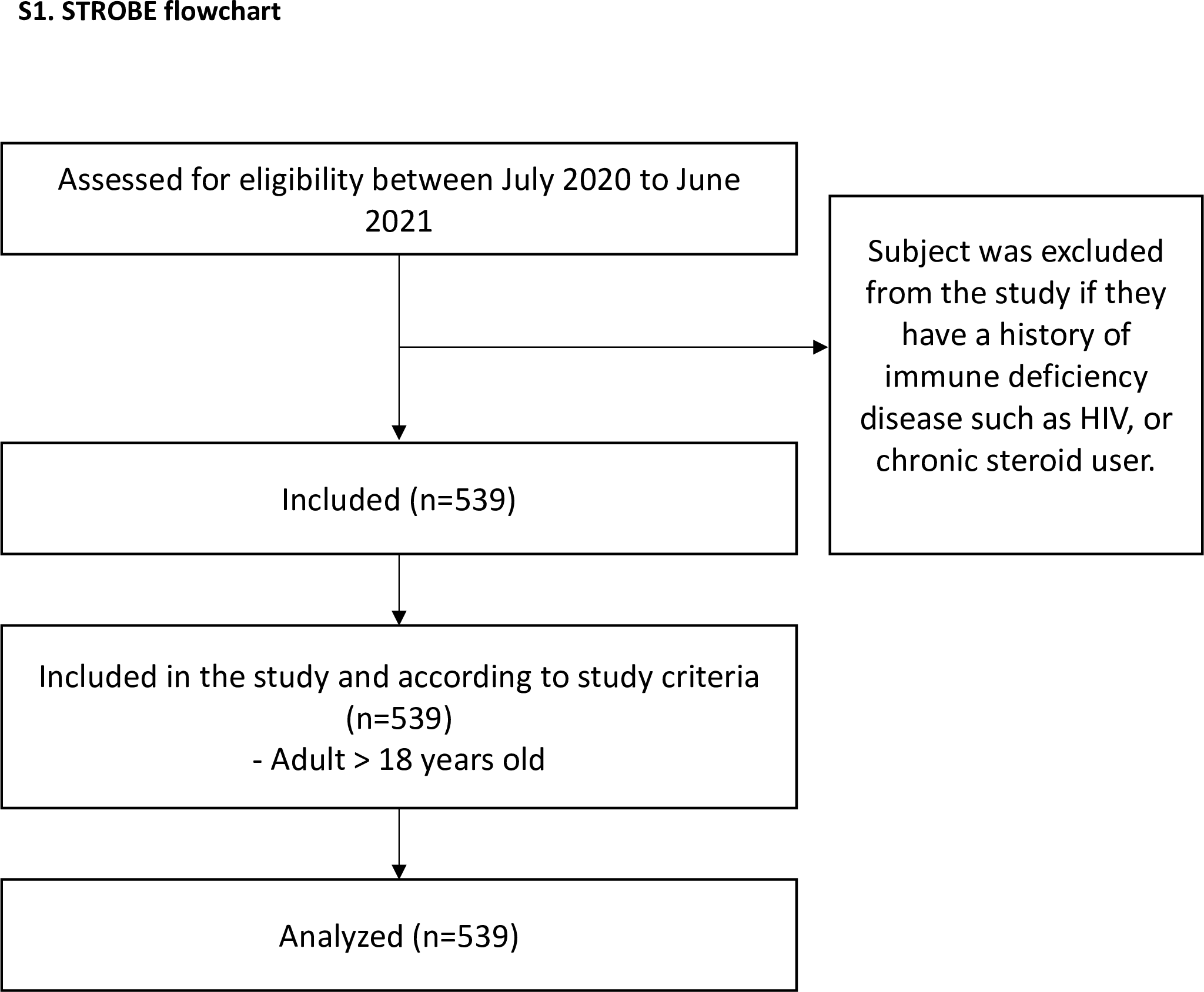

Supplement: S1 Fig — (TIF) [file pone.0271939.s001.tif]
