# Supplementary material for: Dengue seroprevalence study in Bali
Source: PLoS One. 2023 Jul 14;18(7):e0271939. doi: 10.1371/journal.pone.0271939 (PMC10348525; doi:10.1371/journal.pone.0271939)
Supplement: Attachment — Submitted filename: Response to Reviewer-Dengue seroprevalence study in Bali.docx [file pone.0271939.s002.docx]

Dear Honored editor and reviewer,

Please find our answers to each of your comments below.

Thank you so much for your valuable time.

Best regards,

Authors

Reviewer #1: The authors have been concise, which is to be appreciated, but at the expense of explaining how the surveys were done, and that will affect the conclusions that might be reached.

Major points

1) I suggest the authors use the STROBE checklist for reporting of observational studies in epidemiology (von Elm et al BMJ 2007). Please include the checklist in any future version.

**Answer:** Thank you for reminding us. We have already checked using the STROBE checklist and added any information needed.

2) The abstract says: “clinical manifestation of dengue varies from asymptomatic cases to severe dengue manifestation. The detection of clinical cases enables us to measure the incidence of dengue infection”. This is contradictory. Since some cases are asymptomatic, detection of clinical cases will, in fact, underestimate the incidence of infection.

**Answer:** Thank you so much for addressing this. We have already rewritten the sentence and made it clearer.

3) Then the last part of the same sentence is also misleading: “whereas serological surveys give insights into the prevalence of infection.” In fact, serological surveys (as in the current study) are generally for long-lasting antibodies, so are informative about the experience of past infection, not prevalence of current infection, as seems to be suggested. Also, in context, the statement suggests that serosurveys don’t give information about incidence of infection. However, repeat surveys in the same people can do so.

**Answer:** Thank you so much. We have already rewritten the sentence

4) Then the final sentence of the introduction of the abstract is “This study aimed to determine the dengue prevalence among healthy adult patients in Bali.” Given the previous exposition, it’s not clear what’s meant by “prevalence” (i.e. prevalence of what?). Also, how can they be both healthy and patients? (See below about doubts about the population.)

**Answer:** Thank you so much. We have already clarified and rewritten the sentence.

5) In the results section of the abstract, measures of association (e.g. odds ratios) and confidence intervals should be included, not only p values.

**Answer:** We have already added the odds ratios and confidence intervals in the abstracts.

6) In the concluding section of the abstract says: “The current study highlighted a high prevalence of dengue seropositive with a relatively dominant proportion of asymptomatic cases.” The second part is not worth highlighting because the participants are said to be healthy. Although the exact population is not really clear (see comments about methods below).

**Answer:** We have clarified the terms we used to describe the population in the current manuscript.

7) The English should be reviewed by a native speaker. Generally it is comprehensible but there are exceptions e.g. the first sentence of the introduction: “Dengue has been considered the disease of the forthcoming”.

**Answer:** The current revision has already been proofread by English Editing Service editage.com.

8) In the introduction, again there is apparent confusion between epidemiological terms e.g. “The prevalence of dengue virus (DENV) infection has been predicted to cause 390 million new cases arising each year”. And the introduction closes by saying “The study objective is to assess the prevalence of dengue infection detected by Indirect IgG ELISA” which is misleading because IgG is about history of infection, not only recent or current infections. The same issue arises on line 84 of the results: please check throughout.

**Answer:** Thank you so much. We already revised the objective statement.

9) The methods section starts as follows. “Cross-sectional seroprevalence surveys performed in July 2020 – June 2021 among healthy adults and non-dengue patients in Denpasar Bali. Blood samples collected from each of 539 randomly selected samples from urban sites in Denpasar.” This raises several important questions.

**How many surveys were done?** Once

**What was the random sampling mechanism?** Collecting sample was done by consecutive sampling techniques

**What were the inclusion criteria?** We have already added it in the method section.

**Were some or all of the people included more than once?**  No.

**If the aim was to sample healthy people in their houses then why specify non-dengue patients, and how is it that more than a third of the people (per Table 1) were not healthy?**

Thank you so much for raising this issue. Our participants are both adult healthy people and patients. The patients itself comprised of COVID-19, febrile illness and non-febrile patients.

**E.g. is this illness at the time of the survey, or history of illness?** Illness at the time of the survey

Line 110, in the discussion, mentions history of these participants but it was not mentioned before.

**Answer**: We have added the results

10) In the analysis, I suggest plotting the seroprevalence by age, which will give some idea of the force of infection.

**Answer:** Thank you we add a graph explaining seroprevalence by age group (@5 years)

11) Line 96-97: “The current dengue seroprevalence study conducted on adults in Bali reveals that people in tropical countries are at risk of dengue infection”. There should be a more nuanced discussion of the generalizability of the findings.

**Answer:** We have already added some of the discussion.

12) Line 105-107: “The current study also found that participants aged more than 40 years old had a significantly higher proportion of dengue seropositive than participants aged less than 40 (p=0.005). This finding may be related to the risk of Aedes bites being higher than younger age in their life.” The finding of increased seroprevalence with age is completely expected, and the most economical explanation is simply that older people have had the greater time of exposure.

**Answer:** Thank you for clarifying

13) COVID is mentioned in the title but this isn’t prominent in the interpretation. If the pandemic context is important I suggest explaining why and making relevant conclusions; if it’s not important then it doesn’t need to be mentioned in the title.

**Answer:** We revised the title into dengue seroprevalence study in Bali

**Minor points**

14) The results section of the abstract says “Another seroprevalence study reported a lower rate of dengue infection in children in Indonesia”. First, if this is a separate study then it should not be reported in the results section of the abstract of the current one. Then, again, it’s at best confusing to talk about “dengue infection” here, because it was presumably not current infection. It would be better to talk about “proportion seropositive”.

**Answer:** Thank you for your input. We have already deleted and rewritten the sentence in the abstract.

15) “Aedes” should be in italics throughout.

**Answer:** Revised

16) Line 70-71. “The kit’s specificity and sensitivity are 0.988 (95% CI: 0.979–0.993) and 0.892 (95% CI: 0.879–0.903), respectively.” I suppose this is from a previous source, which should then be cited.

**Answer:** Revised

17) Table 2. Correct spelling: “positif” and “negatif”.

**Answer:** Revised

18) Line 100-102: “This study result is in line with another study that report the seroprevalence rate was high (91.5%) across all eight study sites. The reason is the study was conducted on adults and children in urban and rural populations in Thailand”. The ordering is not clear (eight study sites are mentioned before the study country) and stated reason isn’t very explanatory. Overall, I think the message from this section should be that the seroprevalence is high in the current study, but in line with comparable studies from the region and beyond.

**Answer:** Thank you for your input. We have already rewritten the sentence.
