# Supplementary material for: Dengue seroprevalence study in Bali
Source: PLoS One. 2023 Jul 14;18(7):e0271939. doi: 10.1371/journal.pone.0271939 (PMC10348525; doi:10.1371/journal.pone.0271939)
Supplement: Attachment — Submitted filename: Rebuttal Letter Dengue seroprevalence study in Bali 2802.docx [file pone.0271939.s003.docx]

Dear Academic Editor and Reviewer,

We would like to thank you for reviewing our articles. Please find our answer to the concern raised below.

Thank you.

Best regards,

Authors

**Response to the Reviewer**

Reviewer #2: - In lines 44 and 45, authors described that “Four serotypes of DENV (DENV- 1, -2, -3, and -4) have spread rapidly within countries and across regions, causing epidemics” in line 44 and 45.

Could authors describe the prevalence of the serotype of DENV in Bali?

**Our Response: Thank you. We have described each prevalence of the serotype of DENV in the introduction. (line 56-59)**

- Authors described that “The study found 85.5% of the subject was Dengue antibody detected, of which 336 (72.9%) was healthy subject (Table 2)” between line 105 and 107.

I am understanding that most people already experience infection of dengue virus and the authors also mentioned ADE (antibody-dependent enhancement) of infection (between line 142 and 144).

Did authors check FRNT50 in this study?

**Our Response: The study was conducted during the pandemic, we are short in budget and lab resources, so we did not conduct FRNT50.**

Because after first infection of dengue virus, non-neutralizing antibodies to a specificity serotype of dengue virus are produced and these antibodies are harmful such as shock and hemorrhage after others serotypes are infected (authors also mentioned below.

An individual infected with a different dengue serotype for the second time may experience an antibody-dependent enhancement of infection, which means that antibodies from the previous infection serve to disseminate the viral infection and increase viremia [20])

Therefore, I recommend that authors also showed result of FRNT50 in this study.

**Our Response: As we mentioned, the study was conducted during pandemic, we are short in budget and lab resources, so we did not conduct either FRNT or PRNT. We have also mentioned this as a study limitation in the discussion.**

- In line 121, authors described that “This difference may be associated with a lower probability of Aedes bite among children than among adults” and “This finding may be related to the higher risk of Aedes bites than those who are younger” in line132 and 133.

Could you put the reason in this part?

Because I think that the chance of a bite maybe same (or similarly) between children and adults.

**Our Response: The likelihood of infection increases with age. Even in small concentrations, neutralizing antibodies to the same serotype can still be detected.**

Reviewer #3: Manuscript “Dengue seroprevalence study in Bali”

This manuscript by Masyeni et al. reports a study of dengue seroprevalence in Bali. It employed a cross-sectional seroprevalence survey methodology among healthy and ill adult patients in Denpasar, Bali. The authors noted the high prevalence of dengue seropositivity in Bali. I do have some comments/suggestions that I think would help to strengthen the manuscript.

Major comments/suggestions

1. The objective of study (lines 57-59); this research conducted a seroprevalence survey of dengue infection rather than assessing the prevalence of dengue infection, because single IgG antibody indicates a previous dengue infection rather than an acute or asymptomatic dengue infection. Please revise the text to” “to assess the seroprevalence of dengue infection….”

**Our response: Thank you for your suggestion. We have revised the study objective. (line 61)**

2. The authors should detail sample-size calculations, including the healthy, febrile, and non-febrile subgroup.

**Our response: We determine the minimum sample size for our cross-sectional study using the sample size hypothesis test for different proportions, with the first proportion in dengue cases and the second proportion as healthy exposed.**

Where
Za (1-alfa) with alpha 0.01= 2.576

Zb (1-beta) with beta 0.05= 1.96

P1 as case proportion = 70% (the prevalence from previous study in 2018)

P2 as non-case proportion = 50% (an assumption)

p̂= (P1+P2)/2 = 0.6

**n=〖(2.576√(2x0.6x(1-0.6) )+1.96 √(0.7(1-0.7)+0.5(1-0.5) ) )〗^2/〖(0.7-0.5)〗^2**

**n= 9.697/0.04 = 242.43**

3. The authors should provide the study’s eligibility (inclusion/exclusion) criteria.

**Our response: Thank you so much. We have already written the study’s eligibility in the method. (line 66)**

4. How did the authors randomize participants in the healthy, febrile, and non-febrile groups for enrollment? Please clarify the methodology in the manuscript.

**Our response: The sampling method used snowball/consecutive random sampling.**

5. The authors should provide a study flow chart in the manuscript.

**Our response: Thanks for reminding. We have already added as a supplementary figure.**

6. The authors should provide additional relevant data, including educational level, occupation, type of household, behavior risks, knowledge of dengue, etc.

**Our response: Thank you for suggesting. However, the study did not address the issues.**

7. Table 2; the seroprevalence in the healthy group was higher than any other illness group (90.1% vs 75.3%) with statistically significance; the authors should add some discussion for this finding.

**Our response: Thank you for reminding us. We have already added the discussion about this. (line 143)**

8. Conclusions, lines 154-156. “The current study highlighted a high prevalence of dengue seropositive individuals with a relatively high proportion of asymptomatic dengue cases. The study results can guide physicians on the risk of more severe dengue in every dengue infection in tropical countries.” Actually, this study was unable to detect asymptomatic dengue cases, because a single IgG antibody cannot indicate acute dengue infection. Furthermore, the study was not designed to detect risk/associated factors for severe dengue. Please revise the conclusions accordingly.

**Our response: Thank you so much. We have already revised the conclusion of this study (line 167)**

Minor comments/suggestions

1. Line 106-107; “….of which 336 (72.9%) was healthy subject (Table 2).” In table 2, 336 participants (90.1%) were shown as being in the healthy group, so what does the 72.9% refer to?

**Our response: 72.9% refer to the proportion between 336 positive participants of healthy group per total positive participants 461(85.5%) from both group healthy and any illness.**

2. In response to the reviewer’s comments, the authors should inform us what line numberings have been changed in the revised manuscript.

**Our response: We have added line numberings in this letter.**
