# Supplementary material for: Dengue seroprevalence study in Bali
Source: PLoS One. 2023 Jul 14;18(7):e0271939. doi: 10.1371/journal.pone.0271939 (PMC10348525; doi:10.1371/journal.pone.0271939)
Supplement: Attachment — Submitted filename: Rebuttal Letter Dengue seroprevalence study in Bali (2).docx [file pone.0271939.s004.docx]

Dear Academic Editor and Reviewer,

We would like to thank you for reviewing our articles. Please find our answer to the concern raised below.

Thank you.

Best regards,

Authors

**Response to the Reviewer**

Reviewer #2: - Authors described that “The study found 85.5% of the subject was Dengue antibody detected, of which 336 (72.9%) was healthy subject (Table 2)” between line 105 and 107.

I am understanding that most people already experience infection of dengue virus and the authors also mentioned ADE (antibody-dependent enhancement) of infection (between line 142 and 144).

Did authors check FRNT50 in this study?

Our Response: The study was conducted during the pandemic, we are short in budget and lab resources, so we did not conduct FRNT50.

: Could authors put reference (s) about ADE in this region if authors had no data about ADE? Because the ADE issue is very important in Dengue virus infection

**Our Response: We added discussion on line 165-170 about ADE in Indonesia, because until now there isn’t any study that asses dengue ADE in Bali, and subsequent references can be found on line 253-260.**

Reviewer #3: Manuscript “Dengue seroprevalence study in Bali”

According to their responses and the revised manuscript, the authors appear to have addressed the reviewers' comments. However, the manuscript still requires minor revisions before acceptance for publication:

1. The authors should demonstrate their sample-size calculations for the healthy and any illness subgroups.

2. The sample-size calculations should be included in the manuscript.

3. Lines 143-148: discussion of the statistically significantly higher seroprevalence among the healthy group compared with the any illness group needs strengthening. Individuals with a history of immunodeficiency were excluded according to the exclusion criteria. Therefore, individuals with obvious/severe immunodeficiency conditions should not be enrolled into this study. However, if mildly immunodeficient individuals were unintentionally enrolled into the study, they should be represented in both subgroups. Therefore, the authors’ discussion may not make sense. The authors may look for other factors such as age of the participants in both subgroups because seroprevalence of dengue increase according to their older age.

**Our Response:**

**1&2. We added sample-size calculation as well as the formula that were used to determined minimal number of samples required in the study. This information can be found on line 73-80.**

**3. Thank you for your suggestion, based on your input we added new explanation on line 152-155 with references on line 250-252**
